# Asymmetric transfer between the learning of the complex stimulus

**DOI:** 10.3389/fnins.2025.1578862

**Published:** 2025-04-28

**Authors:** Yangyang Du, Hui Kou, Huijie Liu, Taiyong Bi

**Affiliations:** ^1^School of Management, Zunyi Medical University, Zunyi, China; ^2^School of Medical Information Engineering, Zunyi Medical University, Zunyi, China

**Keywords:** perceptual learning, visual working memory, facial emotion, face perception, transfer

## Abstract

**Introduction:**

Perceptual learning of complex stimulus (such as faces or houses) are shown to be specific to the stimulus, indicating the plasticity of the human high-level visual cortex. However, limited understanding exists regarding the plasticity of the representation of complex stimuli in visual working memory (VWM) and its specificity.

**Methods:**

To address this question, we adopted a delayed match-to-sample task to train the working memory for faces and houses. Subjects were trained for 6 days with neutral faces, happy faces, sad faces, and houses in Experiments 1, 2, 3, and 4, respectively.

**Results:**

The results revealed that training significantly increased the sensitivity (d’) to discriminate the visual representations in VWM in all four experiments. Furthermore, the learning effects of neutral faces were transferable to emotional faces and vice versa. However, the learning effects of emotional faces exhibited limited transfer to untrained emotional faces. More importantly, the transfer of learning effects between faces and houses was asymmetrical, i.e., only the learning effects of faces could transfer to houses, whereas the reverse was not true.

**Discussion:**

These results highlight distinct cognitive processes underlying the training effects for different stimulus categories and provide valuable insights into the mechanisms of VWM improvement.

## Introduction

Perceptual learning refers to any relatively permanent and consistent change in the perception of objects and their features (Gibson, 1963). A large number of previous studies have shown that perceptual learning can improve the detection and discrimination of many basic visual features, such as orientation (Du et al., 2023; Wang et al., 2016) and contrast (Roberts and Carrasco, 2022; Yu et al., 2016). Perceptual learning has traditionally been characterized by its specificity to the learned features. For example, Schoups et al. (1995) found that perceptual learning of orientation was specific to the trained orientation and position. In addition to the learning of elementary features, specificity has also been observed in the learning of complex stimuli. For example, facial viewpoint learning was specific to the learned viewpoint (Bi et al., 2010); facial expression learning was restricted to the trained expression (Du et al., 2016).

Although most studies have demonstrated strong specificity in various types of learning, it is important to noted that they frequently employed tasks such as visual discrimination, which demand high precision in the visual processing of a single attribute of the stimulus at the same time. This training paradigm can lead to overfitting and thus specificity (Sagi, 2011). Moreover, previous studies have focused on how training alters the perceptual encoding of the stimuli (Chen et al., 2014; Yan et al., 2014) or the decision-making process (Kahnt et al., 2011; Kuai et al., 2013). However, mnemonic processing also matters for discrimination judgments where the to-be-compared stimuli are often sequentially presented (Jia et al., 2021). Thus, it is essential to investigate the specificity and transferability of learning across a broader range of training tasks, such as the VWM task.

VWM is a crucial cognitive function that involves the ability to maintain and manipulate perceptual information for a short period of time (Baddeley, 1992). Similar to learning observed in perceptual tasks, training has been shown to significantly enhance performance on the VWM task. For example, studies have shown that training significantly increased the accuracy of visual working memory and such a learning effect could partly transfer to a visual working memory task with different stimuli (Bi et al., 2020). However, This study only focused on the learning of the VWM of neutral faces and whether it can be transferred to the house, but did not investigate whether the VWM of emotional faces can be learned and whether it can be transferred between different emotional faces. More importantly, the previous evidence could not discriminate the mechanisms of memory capacity enhancement and perceptual refinement in the learning process.

Therefore, in the current study, we aim to examine whether the learning effects of facial VWM training can transfer to untrained emotions and the same task with houses. Different from perceptual learning tasks, VWM training requires subjects to be based on multiple rough features of the stimulus at the same time (consistent with the resource model of VWM, which posits flexible allocation of resources across features), rather than only on a single fine feature of the stimulus, and these features need to be stored in short-term memory, avoiding specificity caused by overfitting. This aligns with domain-general VWM frameworks that emphasize capacity limits rather than stimulus-specific representations (Cowan, 2001). Therefore, we hypothesize that the learning effects of VWM can be transferred between different stimuli.

## Methods

### Subjects

We conducted a power analysis using G*Power 3.1.9.7 to determine the appropriate sample size. Based on an assumed effect size of *d* = 0.8, a significance level of *α* = 0.05, and a statistical power of 0.95, the analysis indicated that a minimum sample size of 19 participants would be required to detect a significant improvement during training. A total of 80 undergraduate students (42 females) were recruited for the study. Participants ranged in age from 18 to 21 years (M = 19.40, SD = 1.10), with 20 participants assigned to each experimental condition. No individual participated in more than one experiment. All participants were right-handed, had normal or corrected-to-normal vision and reported no history of neurological or psychiatric disorders. The study protocol was approved by the Ethical Committee of Zunyi Medical University and conducted in compliance with the principles outlined in the 1964 Declaration of Helsinki and its subsequent amendments. Written informed consent was obtained from all participants prior to their involvement in the study.

### Materials

Sixty adult facial images (30 female) were selected from the Chinese Facial Affective Picture System (CFAPS), comprising an equal number of neutral, happy, and sad expressions (Yan and Yue-jia, 2005). Hair and ears were removed using Photoshop software. Sixty house images were obtained from online sources. All images were then standardized by superimposing them onto a black background and resizing to 151 × 138 pixels, corresponding to a visual angle of 3.3 × 3 degrees. Subsequently, the images were converted to grayscale and normalized for uniform brightness and contrast using MATLAB (version R2021b; MathWorks).

The visual stimuli were presented on a 19-inch LCD monitor with a spatial resolution of 1,024 × 768 pixels and a refresh rate of 60 Hz. Participants were instructed to maintain fixation on a centrally located white dot (0.2° visual angle) throughout the experimental session. To ensure head stability, a chin rest with an integrated headrest was employed. The viewing distance was maintained at 60 cm.

### Procedure

**Figure 1:**
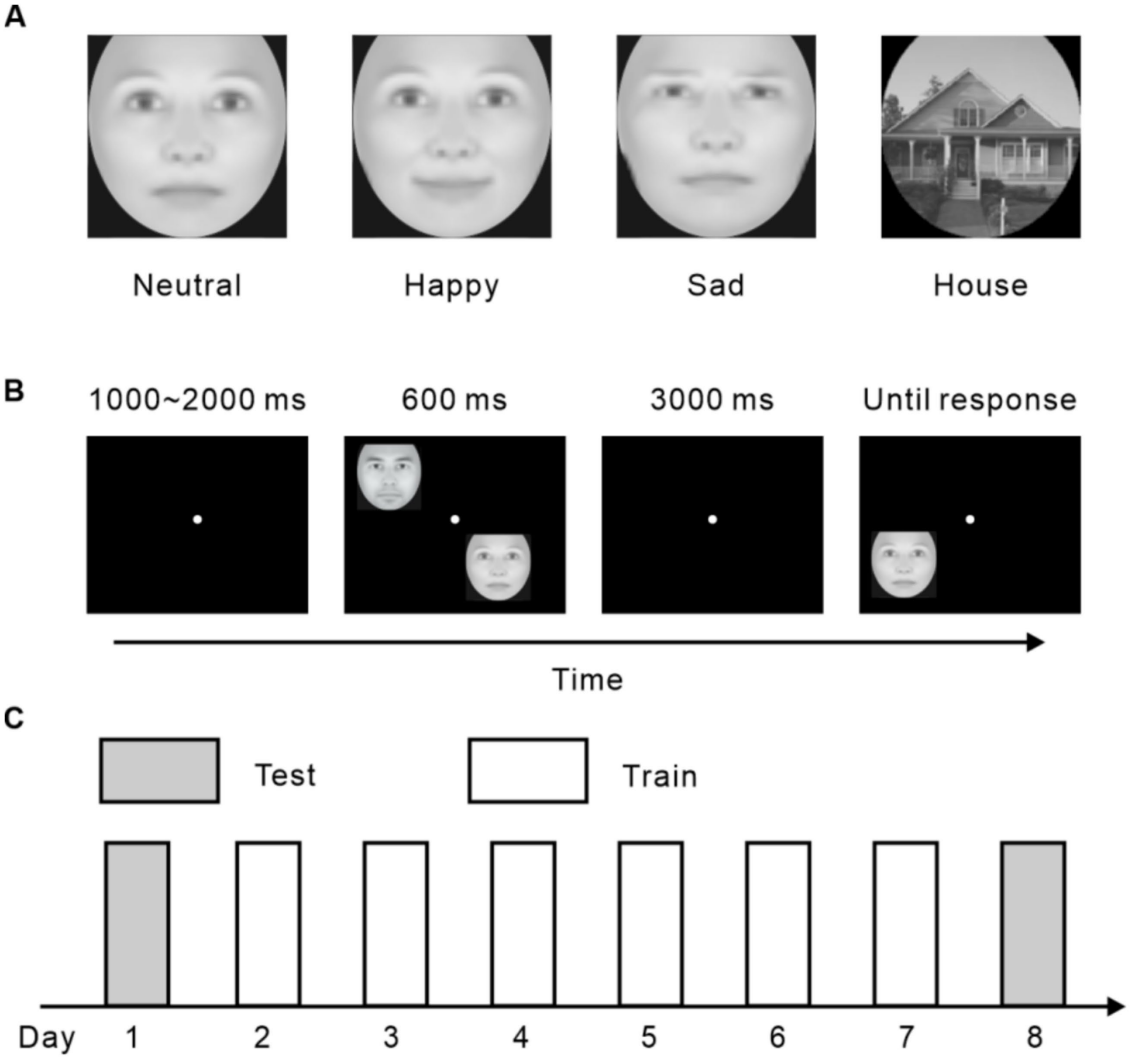
Stimuli and experimental procedure. **(A)** Demonstrations of experimental stimuli. **(B)** A sample trial of the VWM task. **(C)** The protocol of all four experiments.

The current research consisted of four experiments. Subjects were trained to memorize pictures with neutral faces, happy faces, sad faces, and houses in Experiment 1, 2, 3, and 4, respectively (Figure 1A). In all experiments, each participant completed six daily training sessions, each lasting 1 h, along with two test sessions conducted either before or after the training sessions (Figure 1C). Every training session comprised 12 blocks of VWM tasks, with each block containing 40 delayed match-to-sample (DMTS) trials (Figure 1B). At the start of each trial, a fixation point was displayed. Following this, two distinct images (samples), randomly selected from the stimulus set, were presented simultaneously for 600 ms. After the samples disappeared, a blank screen with a fixation point was shown for 3,000 ms. During this interval, participants were required to retain the identities of the sample faces or houses in their memory. Subsequently, a test stimulus was displayed, and participants had to quickly and accurately determine whether it matched one of the previously shown samples by responding “yes” or “no.” If the response was incorrect, a high-pitched sound was played as feedback, and the next trial began immediately afterward. The stimuli were presented at four fixed locations, one in each quadrant of the visual field, with each position positioned 3 degrees of visual angle away from the fixation point. The presentation of stimuli across these four locations was randomized throughout the experiment.

During the test sessions, subjects completed a total of 24 blocks of VWM tasks each day, with six blocks for each kind of stimuli. The procedure was analogous to that of the training sessions except that no feedback was provided.

### Data analysis

The detection sensitivity (d’) (Green and Swets, 1966) was first calculated for each block. Afterward, in the training sessions, We calculated the average d’ across the 12 blocks for each day during the training sessions. For the test sessions, d’ values were averaged across the six blocks for each condition (neutral face, happy face, sad face, and house) per day. To better illustrate the learning and transfer effects, we also computed the performance improvement (∆d’), defined as the difference in d’ for the same condition between the Post-test and Pre-test sessions. Statistical analyses were conducted using repeated-measures ANOVA and one-sample t-tests to compare means across different conditions and groups. To account for multiple comparisons, the Bonferroni adjustment was applied. Additionally, statistical power and effect sizes (Cohen’s d’ for *t*-tests and partial η^2^ for *F*-tests) were reported where appropriate to provide further insight into the results. JASP software (JASP Team, 2021) was used for all statistical tests.

## Results

### Experiment 1

**Figure 2:**
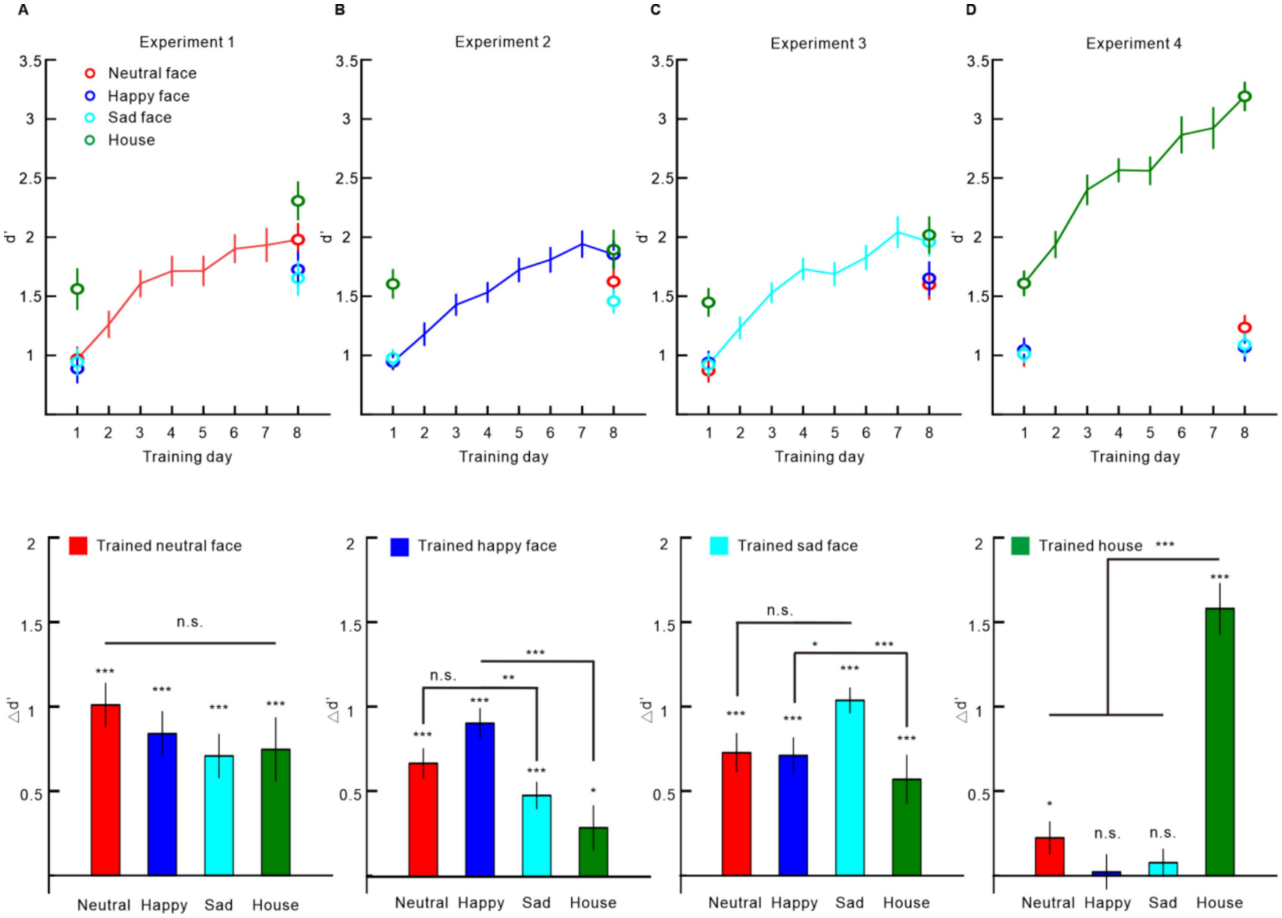
Training effect results. **(A)** Experiment 1 results with neutral faces as training stimuli. **(B)** Experiment 2 results with happy faces as training stimuli. **(C)** Experiment 3 results with sad faces as training stimuli. **(D)** Experiment 4 results with houses as training stimuli. In each experiment, the upper panel displays the d’ during the testing and training phase; the lower panel shows the ∆d’ between post-tests and pre-tests. Error bars represent one standard error of the mean. Statistical significance is indicated as follows: *p < 0.05, **p < 0.01, ***p < 0.001, Bonferroni corrected.

In this experiment, participants underwent training using neutral faces. After the 6-day training, the d’ was improved not only for the directly trained neutral faces [∆d’ = 1.010 ± 0.127, one-sample *t*-test, *t*(19) = 7.974, *p* < 0.001, Cohen’s *d* = 1.783; 95% confidence interval (CI95): 1.061–2.487], but also for the happy faces [∆d’ = 0.839 ± 0.130, *t*(19) = 6.477, *p* < 0.001, Cohen’s *d* = 1.448; CI95: 0.806–2.072], sad faces [∆d’ = 0.707 ± 0.128, *t*(19) = 5.520, *p* < 0.001, Cohen’s *d* = 1.234; CI95: 0.639–1.812%], and houses [∆d’ = 0.746 ± 0.186, *t*(19) = 4.004, *p* < 0.001, Cohen’s *d* = 0.895; CI95: 0.366–1.409] (Figure 2A). The improvements in all the conditions were not statistically different [repeated measures ANOVA, *F*(3, 57) = 1.960, *p* = 0.130, partial η^2^ = 0.094], indicating a complete transfer of learning effect to the untrained stimuli (Figure 2A).

### Experiment 2

In this experiment, participants underwent training using happy faces. After the 6-day training, the d’ was improved not only for the directly trained happy faces [∆d’ = 0.908 ± 0.085, one-sample *t*-test, *t*(19) = 10.649, *p* < 0.001, Cohen’s *d* = 2.381; CI95: 1.503–3.242], but also for the neutral faces [∆d’ = 0.671 ± 0.085, *t*(19) = 7.849, *p* < 0.001, Cohen’s *d* = 1.755; CI95: 1.040–2.452], sad faces [∆d’ = 0.481 ± 0.077, *t*(19) = 6.215, *p* < 0.001, Cohen’s *d* = 1.390; CI95: 0.760–2.001], and houses [∆d’ = 0.289 ± 0.131, *t*(19) = 2.215, *p* = 0.039, Cohen’s *d* = 0.495; CI95: 0.024–0.955] (Figure 2B). However, repeated measures ANOVA showed that the improvements were significantly different among the four conditions [*F*(3, 57) = 11.609, *p* < 0.001, partial η^2^ = 0.368]. *Post-hoc* tests with Bonferroni correction showed significantly higher ∆d’ in happy faces than sad faces (*p* = 0.002) and houses (*p* < 0.001) and no significant difference between the ∆d’ in happy faces and neutral faces (*p* = 0.235) (Figure 2B).

### Experiment 3

In this experiment, participants underwent training using sad faces. After the 6-day training, the d’ was improved not only for the directly trained sad faces [∆d’ = 1.038 ± 0.073, one-sample *t*-test, *t*(19) = 14.265, *p* < 0.001, Cohen’s *d* = 3.190; CI95: 2.085–4.280], but also for the neutral faces [∆d’ = 0.728 ± 0.112, *t*(19) = 6.497, *p* = *p* < 0.001, Cohen’s *d* = 1.453; CI95: 0.809–2.078], happy faces [∆d’ = 0.712 ± 0.103, *t*(19) = 6.894, *p* < 0.001, Cohen’s *d* = 1.541; CI95: 0.877–2.187], and houses [∆d’ = 0.570 ± 0.141, *t*(19) = 4.045, *p* = *p* < 0.001, Cohen’s *d* = 0.904; CI95: 0.373–1.419; Figure 2C]. However, repeated measures ANOVA showed that the improvements were significantly different among the four conditions [*F*(3, 57) = 5.907, *p* = 0.001, partial η^2^ = 0.237]. *Post-hoc* tests with Bonferroni correction showed significantly higher ∆d’ in sad faces than happy faces (*p* = 0.037) and houses (*p* < 0.001) and no significant difference between the ∆d’ in sad faces and neutral faces (*p* = 0.055) (Figure 2C).

### Experiment 4

In this experiment, participants underwent training using houses. After the 6-day training, the d’ was improved for the directly trained houses [∆d’ = 1.582 ± 0.147, one-sample *t*-test, *t*(19) = 10.745, *p* < 0.001, Cohen’s *d* = 2.403; CI95: 1.519–3.270], and for the neutral faces [∆d’ = 0.224 ± 0.094, *t*(19) = 2.398, *p* = 0.027, Cohen’s *d* = 0.536; CI95: 0.060–1.000], but not for the happy faces [∆d’ = 0.023 ± 0.100, *t*(19) = 0.227, *p* = 0.823, Cohen’s *d* = 0.051; CI95: −0.388 to 0.489] or sad faces [∆d’ = 0.077 ± 0.080, *t*(19) = 0.970, *p* = 0.344, Cohen’s *d* = 0.217; CI95: −0.229 to 0.658; Figure 2D]. Repeated measures ANOVA showed that the improvements were significantly different among the four conditions [*F*(3, 57) = 72.981, *p* < 0.001, partial η^2^ = 0.793]. *Post-hoc* tests with Bonferroni correction showed significantly higher ∆d’ in houses than neutral faces, happy faces, and sad faces (all *p*s < 0.001) (Figure 2D).

## Discussion

The current study showed that the training on a VWM task significantly enhanced the VWM performance. Interestingly, such training effects were modulated by the training set of stimuli. First, we observed that performance improvements were not limited to the specific emotion used during training but also extended to other emotions. This transfer effect was consistently demonstrated between happy and sad faces across two independent experiments. Second, we found that training on detecting facial expressions, whether emotional or neutral, resulted in bidirectional transfer between these categories. Since training was exclusively provided for one emotion, the observed improvements in untested emotions provide strong evidence that these transfer effects arise from a shared component between the trained and untested emotions. This suggests a common underlying mechanism linking happy and sad faces, as well as neutral and emotional faces. Additionally, we discovered an asymmetric transfer effect between faces and houses. Specifically, the learning effect for faces transferred to houses, but the reverse was not true—training on houses did not significantly transfer to faces. This asymmetry may indicates that VWM training for faces primarily enhances memory capacity, whereas training for houses mainly refines perceptual representations within VWM. This interpretation aligns with the slot model (Cowan, 2001), which suggests that face training improves the allocation of limited VWM slots to both faces and houses due to shared capacity constraints. In contrast, house training may rely more on feature-based resource allocation (Bays and Husain, 2008), leading to stimulus-specific perceptual refinement with limited transfer. These findings underscore the existence of distinct cognitive processes that drive the training effects for different stimulus categories, offering important insights into the mechanisms underlying VWM improvement.

Using the delayed match-to-sample task, we found that training can improve the ability to recognize facial expressions, consistent with previous studies (Bi et al., 2024; Du et al., 2016; Haller et al., 2022). More importantly, our findings indicated that the effects of training were not restricted to the trained expression. This finding was inconsistent with a previous study adopting a facial expression discrimination task (Du et al., 2016), in which the training effect showed strong specificity to the trained expression. However, some other researches using different task paradigms have found that learning can be transferable between different emotions. For example, a recent study adopting a visual search task revealed strong transfer across expressions, such as neutral, happy, and sad faces (Bi et al., 2024). In addition, another study adopting an expression detection task revealed strong transfer between specific expressions, such as disgust and anger, as well as fear and surprise (Wang et al., 2019). An evident difference among these researches was the task of training. Du et al. (2016) examined the discrimination performance, which requires high precision in the visual processing of a single attribute of the stimulus at the same time, while Bi et al. (2024), Wang et al. (2019) and the present study examined the detection and maintenance of expression, which require low precision in the visual processing of multiple attributes of the stimulus. Taken together, the higher generality of the training effect in our study suggests that VWM training may primarily enhances higher-level cognitive functions, such as memory capacity to facial expressions, rather than lower-level expression processing mechanisms.

However, it is important to note that the transfer effect between happy and sad faces was not complete in our results. This partial transfer could be explained by two potential factors. First, VWM training may exhibit some degree of stimulus specificity, potentially influencing perceptual processing to a certain extent. To test this hypothesis, further research involving neurophysiological studies is needed to provide more direct evidence on how WM training affects the perceptual processing of stimuli. Second, the intensive training may have increased the familiarity of the trained stimuli (e.g., happy or sad faces) compared to the untrained stimuli, making them easier to encode into working memory. This familiarity effect could have contributed to the observed asymmetry in transfer, as the trained stimuli became more efficiently processed over time. Evidence showed that the VWM performance was better for famous faces than unfamiliar faces (Jackson and Raymond, 2008). Therefore, familiarity emerges as a critical factor that may account for the observed differences in performance between trained and untrained stimuli. However, such an effect is not evident in Experiment 1. In this experiment, the learning effect can be completely transferred between familiar and unfamiliar faces (Figure 2A).

Another important and interesting finding was the asymmetric transfer of the training between faces and houses. We found that the learning effect of faces could transfer to houses, which is consistent with previous findings (Bi et al., 2020); but the learning effect of houses could hardly transfer to faces. A typical factor causing asymmetric transfers in perceptual learning is the difficulty of the task. The reverse hierarchy theory proposes that learning can transfer from a simple task to a difficult one but not from a difficult task to a simple one (Ahissar and Hochstein, 2004). However, our results contradict this interpretation. From the results of the four experiments, it is evident that the d’ for face tasks is relatively lower than that for the house task, which indicates that the difficulty of the face task is higher than the house task. Therefore, the mechanism underlying the VWM training may be different from the perceptual learning. We speculate that face training may be object-based, while house training is feature-mediated. This hypothesis is backed by a recent study (Qian et al., 2019). This study revealed that the nature of unit storage in Visual VWM varies depending on the task difficulty.

While our findings suggest that VWM training can induce transfer effects across emotional expressions, several limitations should be noted. First, the use of a homogeneous participant sample (e.g., young adults) may limit the generalizability of results to other populations. Second, the training duration was relatively short, and long-term retention of transfer effects remains unexplored. Finally, our stimuli focused on prototypical emotional expressions; future studies could incorporate more dynamic or ambiguous expressions to better approximate real-world scenarios. Despite these limitations, our findings have implications for both theoretical and applied domains. For instance, VWM training paradigms targeting emotional expressions could be adapted for clinical populations with social cognitive deficits (e.g., autism spectrum disorder) or integrated into cognitive rehabilitation programs to enhance emotional resilience.

## Conclusion

In conclusion, this study employed a delayed match-to-sample task to test VWM for faces and houses. The results suggest that there is a shared learning mechanism between neutral faces and emotional faces. In addition, VWM storage for faces and houses may be object-based and feature-based, respectively.

## Data Availability

The raw data supporting the conclusions of this article will be made available by the authors, without undue reservation.
